# Cushing’s disease

**DOI:** 10.1186/1750-1172-7-41

**Published:** 2012-06-18

**Authors:** Frederic Castinetti, Isabelle Morange, Bernard Conte-Devolx, Thierry Brue

**Affiliations:** 1 Department of Endocrinology and Reference Center for Rare Pituitary Diseases La Timone Hospital, Aix-Marseille University, Marseille, France

## Abstract

Cushing’s disease, or pituitary ACTH dependent Cushing’s syndrome, is a rare disease responsible for increased morbidity and mortality. Signs and symptoms of hypercortisolism are usually non specific: obesity, signs of protein wasting, increased blood pressure, variable levels of hirsutism. Diagnosis is frequently difficult, and requires a strict algorithm. First-line treatment is based on transsphenoidal surgery, which cures 80% of ACTH-secreting microadenomas. The rate of remission is lower in macroadenomas. Other therapeutic modalities including anticortisolic drugs, radiation techniques or bilateral adrenalectomy will thus be necessary to avoid long-term risks (metabolic syndrome, osteoporosis, cardiovascular disease) of hypercortisolism. This review summarizes potential pathophysiological mechanisms, diagnostic approaches, and therapies.

## Disease name and synonyms

Cushing’s disease, corticotroph adenoma, pituitary dependent Cushing’s syndrome.

Chronic glucocorticoid excess, or Cushing’s syndrome, may be due to ACTH-dependent (80% cases) or –independent (20% cases) causes (Table 1). The latter are mainly due to benign (60%) or malignant (40%) adrenal tumors. ACTH overproduction may be of pituitary origin (85% cases) or result from ectopic tumor secretion (15% cases). The term Cushing’s disease is specifically applied to ACTH-secreting pituitary tumors. Cushing’s disease, first described by Harvey Cushing in 1932, represents the most frequent cause of Cushing’s syndrome [1].

**Table 1:** Classification of most frequent causes of Cushing’s syndrome

**ACTH dependent Cushing’s syndrome**	Cushing’s disease or ACTH secreting pituitary adenoma
	Ectopic ACTH secretion
**ACTH independent Cushing’s syndrome**	Adrenal adenoma
	Adrenal carcinoma
	Bilateral adrenal hyperplasia
	Iatrogenic Cushing’s syndrome (Exogenous glucocorticoid exposure)
**Pseudo-Cushing’s syndrome**	Obesity
	Alcoholism
	Depression

Cushing’s disease is defined by Adrenocorticotropin hormone (ACTH) hypersecretion, induced by a corticotroph adenoma, and leading to cortisol hypersecretion (associated with androgens hypersecretion).

## Epidemiology

The incidence of Cushing’s syndrome is estimated to be equal to 1–3 cases per million inhabitants per year, whereas its prevalence is close to 40 cases per million inhabitants. Of note, prevalence of hypercortisolism is thought to be equal to 2-5% of patients with poorly controlled diabetes and hypertension. Female preponderance is generally assumed to be close to 3:1 [2]. Cushing’s disease is an extremely rare condition in children, with a peak in adults in the 3^rd^ or 4^th^ decade. Cushing’s disease leads to death if untreated; it is responsible for increased morbidity and mortality, due to cardiovascular complications, infections and psychiatric disturbances [3,4].

## Clinical and biological characteristics

### Clinical characteristics

Hypercortisolic state may include several clinical signs [5,6]

Obesity: obesity with centripetal fat deposition (face, supraclavicular and dorso-cervical fat pads), facial plethora, rounded face, buffalo-hump

Signs of protein wasting: thin skin, abdominal purple to red and wide cutaneous striae (abdomen, flanks, breasts, hips, axillae), easy bruising, slow healing, muscle wasting (lower limbs muscle atrophy)

Bone wasting leading to osteoporosis (possibly leading to fractures)

High blood pressure

Impaired immune defense mechanisms with increased rate of infections

Gonadal dysfunction and hyperandrogenism: hirsutism (more frequently on the face), menstrual irregularity (oligoamenorrhea, amenorrhea)

Mild to severe psychic disturbances(anxiety, depression, irritability…)

The most frequent sign is obesity: abnormal fat distribution is considered as the most sensitive sign [7]. Evidence of protein wasting (osteoporosis, myopathy) is the most specific sign. Conjunction of both should theoretically allow to distinguish between hypercortisolism and simple obesity. However, the severity of hypercortisolism can be highly variable, which frequently makes the diagnosis difficult. Moreover, hypersecretion profiles can be cyclical, leading to very modest phenotypic signs in some patients (subclinical Cushing’s syndrome) [8]. In most cases, diagnosis depends on a high index of suspicion, rather than a florid clinical phenotype. Of note, none of the signs can allow to differentiate Cushing’s disease from any other etiology of hypercortisolism, except in case of tumor related symptoms such as headaches or visual field defect (in macroadenomas).

### Biological characteristics

Non-specific biological signs may include hypokalemia and impaired glucose tolerance or diabetes. Blood count may show increased hemoglobin, increased neutrophils and decreased lymphocytes or eosinophils.

## Etiopathogenesis

### Characteristics of corticotroph adenomas

Cushing’s disease is frequently due to monoclonal benign and slow growing microadenomas (less than 10 mm) [9,10]. Plasma ACTH (and cortisol) classically lose their physiologic circadian periodicity. They are partially resistant to physiologic stimuli (*i.e.*, glucocorticoids), and do not respond to the normal feedback negative loop. In contrast, corticotroph adenomas are inappropriately sensitive to CRH and AVP. Altered CRH secretion as well as POMC qualitative changes in gene expression were also reported to be involved in the pathogenesis of Cushing’s disease. Cushing’s disease can be more atypical: secretion profiles are sometimes cyclic, with hypersecretion preceding a long period of normal secretion [8,11]. Some corticotroph adenomas are called “silent” as they are clinically and biologically comparable to non-secreting pituitary adenomas: diagnosis is made by the pathologist [12]. Finally, rare cases of aggressive pituitary adenomas or carcinomas have been reported [13]. Whether hyperplasia of corticotroph cells is or not a required initial step before the genesis of corticotroph adenoma remains a matter of debate. The origin of the disease, primary pituitary condition or secondary to an abnormality in the hypothalamus (chronic stimulation by CRH [14]), remains a matter of debate.

### Genetic predisposition

Cushing’s disease can be part of Multiple Endocrine Neoplasia Type 1, due to mutations of the *menin* gene. It is a rare syndrome, transmitted in an autosomal dominant manner, which associates hyperparathyroidism, endocrine tumors, and pituitary adenomas in 20-50% cases. Most of these are somatotroph or lactotroph, but corticotroph adenomas have been described in 5-10% of cases. *AIP* (Aryl hydrocarbon receptor Interacting Protein) mutations have been reported in familial pituitary adenomas: secretion profile is usually somatotroph or lactotroph, whereas very rare cases of CD have also been reported [15].

### Potentially involved molecular mechanisms

Triggering signals leading to Cushing’s disease remain unclear. Oncogenes do not appear to be involved, as somatic mutations are usually not present in corticotroph adenomas cells. Recent studies in mice identified a potential role of loss of function of Brg1 (brahma-related gene 1) and HDAC2 (Histone Deacetylase 2) in the pathogenesis of Cushing’s disease. Both proteins form a complex with the glucocorticoid receptor and the orphan nuclear receptor nuclear growth factor IB (NGFI-B) to repress POMC secretion. Interestingly, about 50% of corticotroph adenomas do not express these proteins anymore. The loss of Brg1 could lead to overexpression of cyclin E, leading to increased cell proliferation and sporadic hyperplasia or tumors. Interestingly, tumors with a loss of nuclear localization of Brg1 seem to be more responsive to anticortisolic drugs *in vitro* compared to the ones with a complete loss of Brg1 oncogene [16,17].

Transcription factors involved in progenitors proliferation and differentiation during pituitary embryogenesis could also be involved in pituitary tumorigenesis. TPIT deficiency is known to result in congenital isolated corticotroph deficiency. Patients with other pituitary transcription factors mutations (*PROP1LHX3LHX4HESX1*) usually present combined pituitary hormone deficiencies including inconstant corticotroph deficiency. As some of these factors are still expressed at adult age, and their role is not precisely known, it could be tempting to speculate on potential roles of an overexpression of these proteins in pituitary adenomas ontogenesis. However, to our knowledge, no mutation of any transcription factor has ever been identified in patients presenting with corticotroph adenomas [18,19].

## Diagnosis

Diagnosis of Cushing’s disease is difficult [20]. Clinical signs and symptoms are often non-specific; no single biological test combines optimal sensitivity and specificity for the diagnosis of hypercortisolism and for the determination of its etiology [21]. Moreover, pituitary and adrenal imaging can sometimes be confusing.

Several steps are needed to first confirm the diagnosis of hypercortisolism and then determine its origin: the first will be to confirm the lack of exposure to exogenous glucocorticoids that induces the same clinical characteristics as Cushing’s syndrome and makes hypercortisolism screening unavailable [22]. In normal subjects, cortisol levels reach a peak at early morning and a nadir < 50 nmol/l around midnight. Patients with Cushing’s syndrome lose this circadian rhythm. As a consequence, early morning ACTH and cortisol values are of poor diagnostic value in the screening methods of hypercortisolism. In contrast a midnight cortisol value > 200 nmol/l is strongly suggestive of Cushing’s syndrome [23]. Evaluation of the circardian rhythm of cortisol is however not recommended as a first line screening method for hypercortisolism.

We will not detail precisely all methods and tests proposed to confirm a diagnosis of hypercortisolism (or Cushing’s syndrome, CS): these criteria have been widely described in recent consensus conferences[6,24]. First line screening methods should include either

24-hour urinary free cortisol, repeated at least twice; values should be above 220–330 nmol/24 h depending on the assays, in Cushing’s syndrome, keeping in mind that normal values can be seen in 8-15% of patients with Cushing’s syndrome [25]

cortisol response to 1 mg-overnight dexamethasone suppression test: cortisol value < 50 nmol/l (< 2 μg/dl) excludes Cushing’s syndrome with high sensitivity (95%) but low specificity [26].

cortisol response to low dose dexamethasone suppression test (0.5 mg dexamethasone every 6 hours during 48 hours): cortisol value < 50 nmol/l (< 2 μg/dl) excludes Cushing’s syndrome with a sensitivity and specificity close to 100% [27].

or late night salivary cortisol [25,28,29] : a cortisol value > 2 ng/ml (5.5 nmol/l) has a 100% sensitivity and 96% specificity for Cushing’s syndrome [30].

Pseudo Cushing’s syndrome is defined by the presence of partial clinical signs of hypercortisolism. It can be induced by chronic alcohol consumption, depression and obesity. Diagnosis between Cushing’s syndrome and pseudo Cushing’s syndrome might be difficult despite the use of previously described screening methods [6]. CRH injection coupled with dexamethasone suppression test, is in favor of Cushing’s syndrome with 90% sensitivity and 84% specificity in the presence of peak cortisol > 580 nmol/l and ACTH > 50 pg/ml [31].

**Figure 1:**
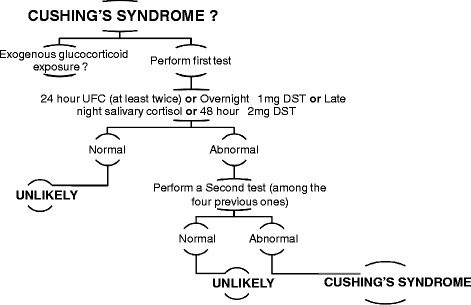
Steps necessary to confirm the diagnosis of Cushing’s syndrome.

Steps for positive diagnosis of Cushing’s syndrome are summarized in Figure 1.

When the presence of CS is confirmed, diagnosis approach will determine if the secretion is ACTH-dependent or not. Early morning undetectable ACTH levels (< 10 pg/ml) will lead to a diagnosis of ACTH independent hypercortisolism (autonomous adrenal hypersecretion), whereas inappropriately normal or increased levels (> 10 pg/ml) will be in favor of an ACTH-dependent hypercortisolism.

ACTH dependent CS includes Cushing’s disease (CD) and, more rarely, ectopic ACTH secretion (EAS) [32]. Distinction between both is difficult, and frequently requires the use of several diagnostic methods [33]:

high dose dexamethasone suppression test (8 mg/day during 2 days): a decrease of more than 50% urinary cortisol level is observed in 90% of patients with CD, compared with less than 50% of those with EAS. Of note, more than 90% suppression of urinary cortisol has 100% specificity in the diagnosis of Cushing’s disease [2].

CRH test (100 μg intra-venously): more than 50% ACTH and 20% cortisol increase is in favor of Cushing’s disease. Sensitivity and specificity are close to 90% [34].

Desmopressin test (10 μg intravenously), ACTH and cortisol increases similar to those observed with the CRH test are in favor of CD with 70% sensitivity and 85% specificity [20,35]

Concordant responses to at least 2/3 of these tests should lead to the diagnosis of Cushing’s disease, and pituitary MRI. However, the sensitivity of MRI in CD is hardly greater than 60-70% and specificity close to 85%, as most corticotroph adenomas are microadenomas. In one study, 10% of the general population presented MRI pituitary images of less than 5 mm that might be considered as adenomas [36]. Cushing’s disease diagnosis is thus confirmed in the presence of an adenoma > 6 mm and concordant responses to tests.

**Figure 2:**
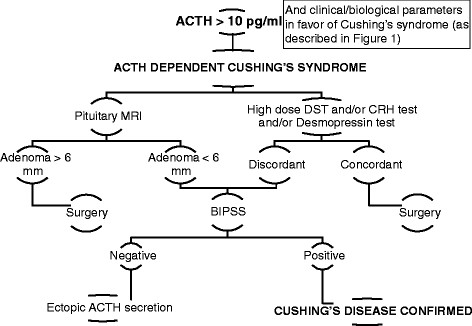
Steps necessary for the etiological diagnosis of ACTH-dependent Cushing’s syndrome.

In the lack of an image suggesting a pituitary adenoma on MRI despite dynamic tests in favor of CD, or in case of discordant tests, bilateral intra-petrosal sinus sampling (stimulated by CRH or desmopressin) should be performed: it will give a definite answer to confirm the etiology of ACTH dependent CS. The principle is to measure a ratio defined by central ACTH/peripheral ACTH. A central to peripheral plasma ACTH ratio exceeding 2 (or 3 after stimulation by CRH) is in favor of Cushing’s disease[37-40] .In case of ACTH dependent hypercortisolism, if the initial etiologic workup is not in favor of a pituitary origin, complementary morphologic investigation including tomodensitometric whole body examination should be performed. Some teams prefer to perform systematically a thoraco–abdomino-pelvic scan in each patient with ACTH dependent Cushing’s syndrome, whatever the status of tests and MRI. The limit of this approach is identical to the one reported with pituitary MRI, as some patients might have bronchial incidentalomas not responsible for ectopic ACTH secretion, and leading to a misdiagnosis [33]. Steps necessary for the etiological diagnosis of ACTH dependent Cusging’s syndrome are summarized in Figure 2.

## Differential diagnosis

Chronic exogenous administration of glucocorticoids

Pseudo-Cushing states as described previously

ACTH dependent Cushing’s syndrome: Ectopic ACTH secretion (see above)

ACTH independent Cushing’s syndrome will be ruled out by inappropriately normal or increased ACTH levels.

Functional hypercortisolism during pregnancy

## Clinical management

Transsphenoidal surgery is the first line treatment of Cushing’s disease[41,42]. It allows remission in 60-90% of microadenomas, and 50-70% of macroadenomas, depending on local invasion and the experience of the neurosurgeon[43-45]. Remission should be defined by normal ACTH and cortisol circadian rhythms, and suppressed cortisol value after overnight/low dose dexamethasone suppression test.

The appropriateness of surgery in the lack of visualized pituitary adenoma remains a matter of debate [46]. When extensive samplings and dynamic tests confirm that hypercortisolism is due to Cushing’s disease, and pituitary MRI seems normal, literature data report a range of surgical efficacy varying from less than 50 to 70% of remission, often associated with induced hypopituitarism and/or diabetes insipidus. The risk of late recurrence after presumably curative surgery is estimated to be close to 25% [47]. Several criteria have been reported as predictive factors for long-term remission: low immediate post-surgical early morning cortisol/ACTH levels, cortisol suppression after 1 mg overnight dexamethasone suppression test, lack of cortisol/ACTH response to desmopressin or coupled dexamethasone desmopressin test [48-51]. However, it is still difficult to predict which patients are at greater risk of recurrence, as some patients uncured immediately after surgery, might however present delayed remission [52]. As a consequence, and due to the high risk of recurrence, it seems difficult to talk about “cure” in patients with surgically treated Cushing’s disease; the term “remission” seems more appropriate. In other words, even long-term remission after surgery should lead to at least a prolonged clinical close follow-up.

In case of immediate surgical failure or late recurrence, several therapeutic modalities are available: second pituitary surgery, medical treatments, radiation techniques, or bilateral adrenalectomy[53]. Only some of these treatments (surgery, and radiation techniques after à prolonged period) can lead to long-term remission.

### Second surgery

Several teams reported the benefits of a second surgical procedure, either in the first days following initial surgery, or later. A recent study reported a possibility of delayed remission after initial surgery in about 5% of cases. This should be in favor of a delayed rather than an immediate approach. Efficacy is usually observed in 50-70% of cases, frequently associated with an increased risk of hypopituitarism, diabetes insipidus, and cerebrospinal fluid leak [54,55].

### Medical treatments

Medical treatments aim at decreasing synthesis and secretion of cortisol, blocking glucocorticoid receptors, or inhibiting ACTH secretion. The main drawback of these drugs is that they are only suspensive, *i.e.* hypercortisolism may be controlled but still uncured, requiring a long-term period of treatment. There are 4 main indications of medical treatment: in case of contra-indication or refusal of surgery, in the lack of adenoma image on pituitary MRI, waiting for radiation techniques to be effective, as a multimodality approach in the rare cases of pituitary carcinomas.

#### Steroidogenesis inhibitors

· Op’DDD (mitotane, Lysodren®) is derived from insecticide dichloro-diphenyl- dichloroethane (DDD). Op’DDD inhibits side chain cleavage of cholesterol and also other cytochrome P450 enzymes (11-alpha and 18-hydroxylase) and non-P450 enzyme (3 beta-hydroxysteroiddeshydrogenase). In Cushing’s disease, it is used as an inhibitor of cortisol secretion.Op’DDD is usually effective in more than 50% cases, and frequently induces adrenal atrophy; however, gastro-intestinal tolerance is usually bad, and there is a 4-week delay to obtain maximal efficacy due to its accumulation in adipose tissue. There is a narrow difference between efficacy and toxicity levels. Main side effects are digestive (nausea, vomiting, diarrhea), neurologic (sleepiness, asthenia) and metabolic (hypercholesterolemia). Mitotane modifies metabolic clearance of steroids with consequent gynecomastia in men and alteration of contraceptive effects of pills [56,57]. Pregnancy is forbidden during mitotane therapy and for two years after drug withdrawal due to teratogenic effects [41,58,59].

· Ketoconazole is an antifungal agent with steroidogenesis inhibitor effects linked to inhibition of cytochrome P450 enzymes. It was reported to normalize cortisol levels in Cushing’s disease in about 50% of cases. Side effects include rare severe liver injury (1/15000 cases), and gastro-intestinal intolerance [60].

· Metyrapone (Métopirone®) is a pyridine derivative that blocks cortisol synthesis by mainly inhibition of 11 beta hydroxylase. Metyrapone is rapidly effective in about 50% of hypercortisolic states: it usually induced low blood potassium levels, and hyperandrogenism [61].

· Etomidate (Hypnomidate®) is an intravenous anaesthetic agent. It inhibits cortisol synthesis by inhibiting CYP11B1 with 11-beta hydroxylase activity, and cytochrome P450scc at high concentrations. Etomidate is a very potent anticortisolic drug, limited by the fact that it can only be used intravenously: it should thus be reserved for severe hypercortisolic states [62].

#### Glucocorticoid receptor antagonist

Mifepristone is currently the only available glucocorticoid receptor antagonist. Only rare cases have been reported to date. The drug seems to be highly effective in controlling clinical signs of hypercortisolism. However, due to its mode of action, there is a high risk of hypokalemia, and there is no biological means to monitor the patient. Treatment dose adjustment is thus only based on subjective signs [63].

#### ACTH-lowering agents

Cabergoline is a dopamine agonist well known for its anti-secretory and anti-tumoral efficacy in prolactinomas. Corticotroph adenomas can express dopamine receptors. Recent studies reported that about 25% of patients treated by high doses of carbergoline for CD could be controlled as well [64-66]. A strict echocardiographic follow-up is required, due to a dose-dependent risk of valvulopathy.

Pasireotide is a somatostatin agonist with a particular binding affinity for somatostatin receptor (sstr) isoforms 1, 2, 3 and 5. This specific affinity for sstr5 could be of major interest in CD. Clinical trials are ongoing to determine efficiency of this drug. Preliminary results suggest that pasireotide is able to decrease cortisol levels in the majority of patients, but only few reach normalized values. There is a risk of induction or worsening of hyperglycemia in 1/3 cases [67-69].

### Radiation techniques

Radiation techniques have been widely used as a treatment of Cushing’s disease. Different techniques are available, mainly based on fractionated radiotherapy or stereotactic radiosurgery. Radiotherapy induces remission in the majority of cases, but also panhypopituitarism in more than 80% of patients [70]. Due to the slow decrease of ACTH levels, delay to remission can vary from 2–3 to 10 years, depending on initial hormone levels. Stereotactic radiosurgery is delivered in a single session. It is theoretically a more precise technique, leading to a lower risk of hypopituitarism. However, the rate of remission is lower, reported in only 50% of cases. Radiosurgery should thus be reserved for small and low secreting lesions [71]. For both techniques, medical treatments need to be given between the procedure and maximal efficacy, *i.e.* for 2–5 years, to control cortisol hypersecretion waiting for normalization.

### Bilateral adrenalectomy

It can be used either in case of failure of pituitary surgery, or when hypercortisolism is severe, requiring a rapidly active treatment. Bilateral adrenalectomy resolves cortisol hypersecretion in the vast majority of cases, with a low risk of perioperative complications [72]. One might consider trying to decrease the level of hypersecretion by antisecretory therapy for a short period of time before bilateral adrenalectomy, but this specific point has never been really evaluated. The major and expected side effect of bilateral adrenalectomy is adrenal insufficiency. Another possible adverse effects is Nelson’s syndrome, which is a pituitary tumor progression observed after adrenalectomy [73].

### Temozolomide

In rare cases, CD can be induced by aggressive pituitary tumors, and still more rarely by pituitary carcinomas. Due to frequent recurrences, they usually require repeat surgery, and aggressive treatments. The rarity of cases makes it difficult to define a consensual therapeutic approach. Recent studies pointed out the benefits of temozolomide, but prospective long-term studies will be necessary to ascertain this point [74-76].

## Prognosis

The risks of chronic hypercortisolic state include excess morbidity and mortality due to increased cardio-vascular risk factors (hypertension, dyslipidemia, diabetes mellitus, metabolic syndrome) leading to heart defect. Moreover, hypercortisolism is responsible for coagulopathy [77] and atherosclerosis [78], which also increase the risk to develop cardiovascular diseases. Recent data suggest that part of these defects due to hypercortisolism might remain after remission [78] even if the mortality rate would go back to normal [79]. Frequency of infectious diseases is also increased, as well as delayed healing. Hypercortisolism can induce severe osteoporosis in about 30% cases, and osteopenia in half of them. Also, acute cortisol excess can induce severe hypokalemia, as well as elevated blood pressure levels, and sometimes psychiatric signs [26,80]. Finally, more than half of patients with CS can present with psychiatric signs, from mild to severe depression, and cognitive dysfunction [81].

## Future prospects

Future research should focus on

1. The pathophysiological mechanisms leading to corticotroph adenomas. Only rare data are available on the early events leading to pituitary adenomas in general, and corticotroph adenomas in particular.

2. The way to improve sensitivity/specificity of diagnostic methods for subclinical Cushing’s syndrome. Some patients are diagnosed with Cushing’s syndrome several years after a prolonged treatment for hypertension, osteoporosis…

3. The way to diagnose earlier patients with a high risk of post-surgical recurrence. About 20% of patients with what is supposedly the best predictive factor of remission (low cortisol levels immediately after surgery) still present long term recurrence of their disease.

4. The development of safe and effective medical treatments. Currently available drugs are either poorly effective and/or have bad tolerance.

## Abbreviations

CS, Cushing’s syndrome; CD, Cushing’s disease; EAS, Ectopic ACTH Secretion.

## Competing interests

The authors declare that they have no competing interest.

## Authors’ contributions

The authors contributed equally to this review. They read and approved the final version of the manuscript.
